# Dentists´ and Dental Hygienists´ experiences of the Capitation Contract System—the dilemma of conflicting loyalties

**DOI:** 10.1038/s41405-022-00110-y

**Published:** 2022-06-20

**Authors:** Emelie Boberg, Bengt Franzon, Annsofi Johannsen

**Affiliations:** 1 Karolinska Institutet, Department of Dental Medicine, Division of Oral Diseases, Huddinge, Sweden; 2 Malmö Universitet, Faculty of Odontology, Department of Oral Diagnostics, Malmö, Sweden

**Keywords:** Dental ethics, Dental patient assessment

## Abstract

**Objective:**

The Capitation Contract system (CCS) is a payment model adopted by the Swedish Public Dental Health Service (PDHS). Patients enrolled in the CCS are usually assessed as being at lower risk of dental disease and are more regular dental attenders than those treated by Fee for Service (FFS). With increasing numbers of patients and CCS enrolments, Sweden faces a shortage of dental personnel. Our aim was to analyse dentists´ and dental hygienists´ perceptions and experiences of the capitation contact system.

**Material and methods:**

Eleven dentists and dental hygienists from three Swedish regions participated in online qualitative interviews conducted according to the Grounded Theory methodology.

**Results:**

When working with CCS the informants tried hard *´to find a balance between attitudes, compliance with guidelines and clinical resources*´. Not all patients were offered CCS, even though they qualified: among other determinants were the informants’ interpretations of guidelines and regulations, clinical resources, and patient interest.

**Conclusions:**

When dental resources are in balance, the informants appreciate the CCS and consider it to be favourable to patient health but are aware of conflicting loyalties of their dual roles of insurance sales agent and care provider. The informants´ individual mindset affects which patients are offered CCS enrolment.

## Introduction

Capitation is a payment system commonly used to cover primary care in health and medical services. It may be tax-based or have a combination of tax and out-of-pocket payment by patients [1, 2]. The health care providers are remunerated with a fixed amount, which is determined by the number and types of patients they are required to treat. This enhances the potential for treating a greater number of patients than under the Fee for Service system (FFS), whereby the patient pays out-of-pocket expenses per treatment [1–3]. There are similar capitation systems in dentistry, as Denplan in Great Britain, Medicaid in the US, and the children and adolescents dental care in Sweden [3–5]. Studies show that patients enrolled in a dental capitation contact system (CCS) are less frequent dental attenders, undergo fewer restorative treatments, and tend to be treated at a later stage than those in the FFS [2]. This may be a result of the care providers remuneration of patients [6]. In many countries, the fixed amount revenue per CCS patient is directly remunerated to the care provider responsible for the risk assessment result but regardless of the treatment outcome. Hence, to enroll healthy patients may enhance the probability to see more patients and elevate the production. It may also increase the risk for undertreatment and patient selection [6].

In Sweden, oral health care is available at small privately-owned dental companies, large privately owned dental companies, and public dentistry (PDHS and PDHS limited). The patient receives free dental care until the year they turn 24 and have to pay for their own dental care, either by the FFS system or the CCS. Both systems are financed by the patient and by subsidies from government-funded dental care support. Children and adolescents receive oral health care free of charge, a type of capitation system financed by the regions [3].

The nationwide start-up of CCS began in 2008 [3] and is almost exclusively offered by the Swedish Public Dental Health Service (PDHS) as an insurance-like payment model remunerated to the PDHS and not a specific care provider. The number of patients enrolled in the CCS has increased continuously: from 30,000 initially to 790,000 in 2020 [7]. This is approximately 20% of the adult population of Sweden [7]. However, about 60% of all adults are registered with private practitioners, whereas most children and adolescents are registered within the PDHS [8]. Pälvärinne et al. [9] showed that PDHS stakeholders regard the CCS as a key to stable economy for the company as it enhances the enrolments of young patients to stay within the company to low costs. Holmes et al. [10] report that English dentists have a target of generated enrolments but mention that this may be counterproductive considering the quality of the provided dental care.

The three-year-long CCS enrolment includes regular check-ups and fixed fee dental care as required, along with patient-specific oral health care recommendations. The patient costs for the CCS cover the predicted need of dental care [3, 11–13], and the financial risk for the PDHS is solved within the organization, because of the large number of enrolees [3]. The price range for CCS enrolment is based on risk assessments, carried out by the dental health care provider at a baseline examination. Peterson & Twetman [14] found that patients assigned to a higher risk group chose not to enroll, while patients assessed as low risk did. However, various types of risk assessments programs are used within the PDHS regions [15]. The Swedish Quality Registry for Caries and Periodontal disease (SkaPa) [15] conclude that the outcome cannot be compared, especially for low-risk groups.

The enrolled patients view the CCS as something that can protect them from unexpected costs, due to the fixed fee, as well as to avoid oral health impairments [16, 17]. Nevertheless, studies have shown that this might also lead to some individuals taking higher risks regarding their oral health care routines [17, 18]. Communication between the caregiver and patient is therefore vital. However, studies show that some patients were unaware or did not comprehend that personal commitments were included in the CCS agreement or know how they were assigned a specific risk group [10, 16, 17].

CCS enrolments can to some extent be linked to the patients’ socioeconomic background [19–22]. Young healthy patients are more likely to enroll [19–21]. Furthermore, only a minority of people from lower socioeconomic groups or elderly patients are aware of the existence of CCS [19]. This may be the result of enrolment recommendations which are included in risk assessments at the base- line examinations [23]. These recommendations, regulating which patients are offered enrolment, are not always highlighted in studies: enrolment is regarded solely as the patient´s own choice [11, 16, 17, 20, 21, 24].

Zickert et al. [24] studied the pilot capitation payment plan carried out in Gothenburg between 1991–1997 and saw that most patients favoured the CCS. They showed greater knowledge of oral self-care and 56% changed their oral hygiene routines for the better.

At the CCS start-up in 2008, dental personnel supply and demand were more or less in balance [25]. At the same time as the number of patients within the CCS continues to increase, demand for dental personnel has become disproportionate, due to large numbers of retirements [25]. Apart from the patients’ own choice, this has further exacerbated the pronounced variations in dental attendance nationwide [8, 19, 26, 27]. It is of interest to note that dental attendance by CCS patients is higher than those in FFS [12, 14]. In addition to this, the CCS patients are more satisfied with their payment plan than the FFS patients [16, 21, 23].

Hence, the aim of this study was to analyse dentists´ and dental hygienists´ perceptions and experiences of the capitation contract system, in the context of their daily routines as care providers.

## Material and method

This semi-structured interview study was conducted in accordance with the constant comparative method of Grounded Theory: [28–30] this method allows construction of theories to understand psychosocial processes and how people act and react in certain situations.

Information about the proposed study was sent via email to Public Dental Health Services (PDHS) in five regions in Sweden. Three regions, Värmland, Västra Götaland and Uppsala, consented to participate in the study. Stockholm and Örebro regions were also contacted but declined the invitation to participate. After approval, contact was mediated with clinic managers from both urban and rural areas from each region: they were informed about participation in the study and informed consent. The clinical manager contacted dentists and dental hygienists at the clinics and informed them about the study and how they could then register their interest. Thereafter, the author (EB) contacted the informants individually and again informed them both written and verbally, about the study and informed consent. Interviews were scheduled and the informants were able to make inquiries about the interview process.

Eleven informants participated: 8 women and 3 men, employed as dentists (*n* = 6) and dental hygienists (*n* = 5) within the PDHS. Two of the participants were clinical managers, some had experience of being responsible for the CCS at their clinic. The work experience was relatively balanced between those who had worked in their profession for a shorter number of years as well as for over 15 years. To ensure that the informants were well acquainted with the CCS, it was a requirement for inclusion in the study that the informants had been employed by the PDHS for at least 3 years.

An interview guide with specified categories of interest was used when conducting the interviews, asking open-ended questions inspired by Charmaz [28]. The ambition was to collect data about the informants’ experience, knowledge, and perceptions of the incorporation of CCS in their daily work. Their responses were used as a guide for relevant follow-up questions. The interview guide categories were (I) work environment, (II) payment models (III) patient health, (IV) cooperation between clinics and stakeholders. The interviews lasted between 30 to 60 min and were recorded as audio files. Because of travel restrictions associated with the Covid-19 pandemic, the interviews were conducted online. New interviews were conducted until saturation was reached [28]. The interviews were anonymized and transcribed by the author (EB).

**Fig. 1:**
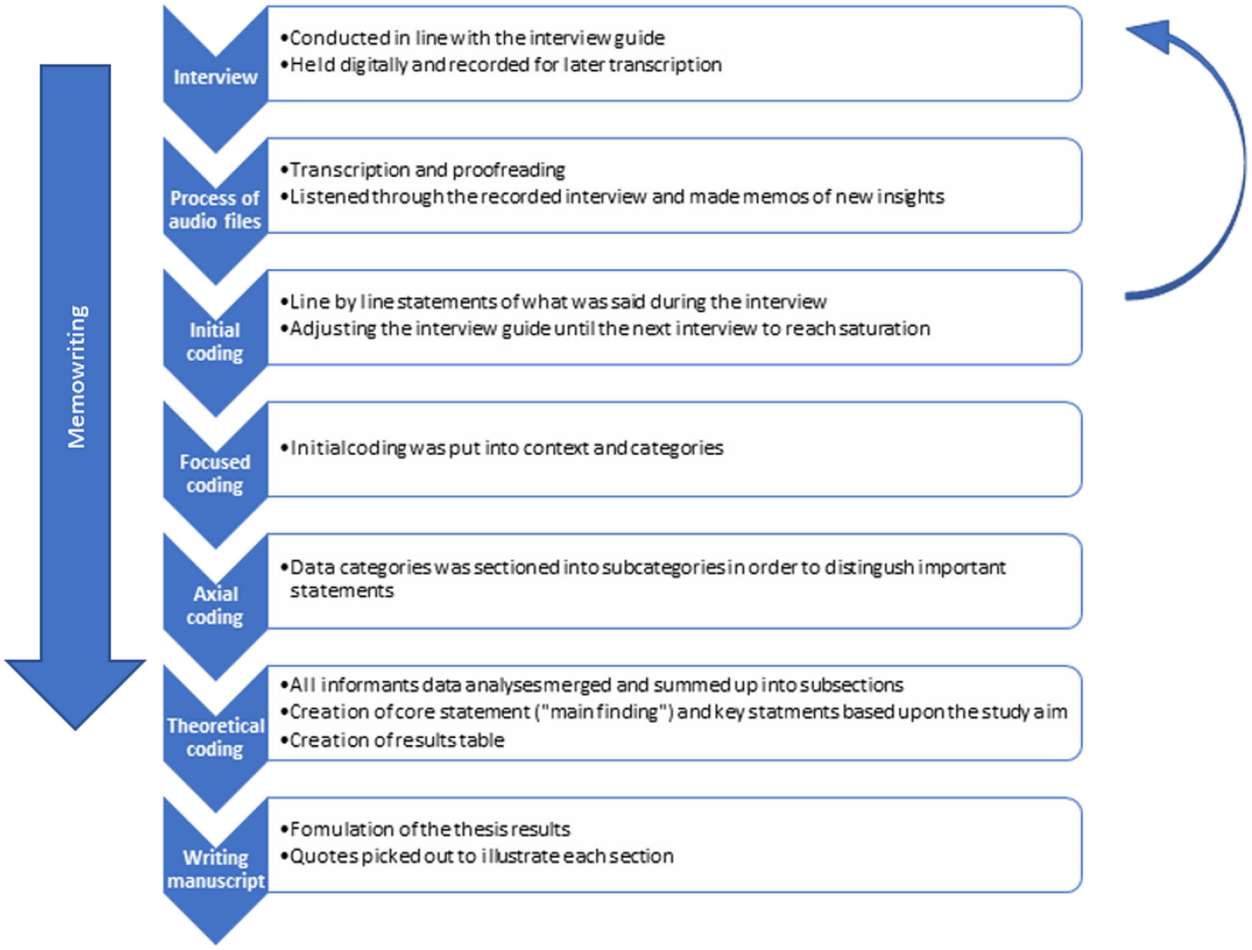
The analytic process. Initial coding adds depth before new interviews take place. The authors’ aspects of the content are written as memos along the process and adds up to the final result of a core statement and supportive key statements.

The data analysis consists of a coding process and memo-writing by the author. The analysis process started when the first interview was read through and was done according to Charmaz [28] who describe this process as four following processes (Fig. 1). These are: (I) initial coding, line-by-line coding which helps to distinguish statements and actions, adds depth and additional approaches to later interviews, (II) focused coding, which creates concepts and categories of the initial coding, (III) axial coding, which organizes the data and specifies the dimensions of the categories and (IV) theoretical coding, which describes possible relationships between the categories and strives to conceptualize a theory called “core statement” and subgroups called “key statements” [28, 31]. The author EB conducted the analyses in close collaboration with the other authors. The transcribed data were read through, and ideas, assumptions, and reflections were written down as memos. Thereafter, descriptive quotes were sorted out to explain the categories and themes which emerged: these were then presented in the results.

## Results

The study revealed that some clinics offer and enroll more patients into CCS than others. The informants attributed this to the socioeconomic status of the area, dental personnel resources and the patient’s own interest, and the ability to enroll.

Moreover, it emerged that the informants’ attitudes towards the CCS system and the PDHS guidelines could affect how and if they offered patients enrolment at all. The informants state that they experience a conflict of loyalties: they are expected to assume the responsibilities of both a caregiver and a CCS promoter. This resulted in the core statement *´to find a balance between attitudes, compliance with guidelines, and resources´* (Table 1).

**Table 1:** Core statement and supportive key statements of the results.

´To find a balance between attitudes, compliance with guidelines and resources´	
***Influence of experience and attitudes on decision making***	• *Financial impact.* • *Oral health knowledge and commitment*
***Impact of compliance with guidelines and regulations as a clinician***	*• Decision-making and experience.* • *Conducting risk assessments.* • *Charging patients for treatments*
***Reasons for inequality in access to oral care***	*• Imbalance of patient volumes and dental staff resources.* • *Recall procedures*

The first key statement was ´Influence of experience and attitudes on decision-making´ with its subcategories; *Financial impact* and *Oral health care knowledge and commitment*.

The second key statement was ´Compliance with guidelines and regulations as a clinician´ with its subcategories *Decision-making and experience, Conducting risk assessments* and *Charging patients for treatments*.

The third key statement was ´Reasons for inequality in access to oral care´ with its subcategories *Imbalance of patient volumes and dental staff resources* and *Recall procedures*.

### Influence of experience and attitudes on decision-making

When making decisions in regard of the CCS, informants recall some aspects that influence their actions and perception of the concept. The difficulty to mind financial aspects of both company and patient matters were brought up to illustrate a contradictory dilemma. Additionally, the informants recall patients’ compliance to recommended oral health care as vital for both initial enrolment and continuity of the CCS. However, they see differencing factors within patient groups which affects how enrolments are executed.

#### Financial impact

The informants experience a dilemma with respect to who will benefit from the enrolment of the patient in CCS. This increases the probability that CCS will be offered to patients in the lower-risk groups: the PDHS benefits from such contracts. On the other hand, the informants stated that these patients do not always receive dental care to the extent covered by their actual fixed payment.

*“Either you focus on the patient’s wallet and perceive it to be too expensive or you focus on the clinic’s finances and think it will be too expensive for the clinic. … I do believe that the patients within the highest risk groups would benefit most from enrolment. But to be honest, the majority of the lower risk group patients do not profit financially from their enrolment*.”

The informants considered that patients were satisfied with their CCS enrolment but cautious about the costs. They also claim that patients are more likely to continue with the CCS if their need for dental care increases but resign if they find their oral health status to be good or if the patient considers the cost to be too high in relation to their contribution. This is typical when the risk assessment results remain unchanged.

*“Pricing is fixed for three years … but that means that the yearly fee adjustments of the pricelist accumulate … and if you are in the same risk group and the fee goes up 10 percent after 3 years, this may seem too expensive”.*

The same informant also added that the CCS adds a greater overview of costs and personal budget for the patient in comparison to the fixed fee payments.

*“The patients should understand that if they have a greater need for oral health care, the better the reason to sign a contract. Because then they can keep track of their expenditures. It is an unpleasant surprise when something needs tending: “there goes 10.000 SEK, which I could have saved for a vacation”.*

#### Oral health care knowledge and commitment

With respect to dental attendance, the informants noted a difference between risk groups and the contributing factors. The low-risk group patients want to visit the dentist relatively more often than warranted by assessed need. The opposite is seen in the high-risk groups. Periodontal disease was thought of something that would increase the risk group but also that the prevalence of healthy patients would decrease in relation to increasing age.

*“There will always be patients that never achieve a healthy periodontal status. And there will always be reasons for us to treat they continuously throughout their lifetime”.*

A good oral health status is a precondition for patients to be part of the CCS. However, the informants recall that patients expect to be cured when undergoing dental treatment. Consequently, informants express a need to improve their communication of risk and responsibility distribution. They also note that some patients do not regard self-care routines as important for lowering the risk of developing disease in future. One example mentioned is that concern about caries is low.

*“To be honest, people in 2020 do not need to get dental cavities. It is in fact really a matter of information. And we try hard to spread the message that this is not normal [to get cavities], it is a disease. And we have tried this for a long time now, however with a low success rate”.*

### Compliance with guidelines and regulations as a clinician

There are several guidelines that care providers need to follow in order to provide oral health care in the means of CCS. How these guidelines are being put into practice is found to be based on individual perceptions. Informants consider that work-life experience, their relation to patients, and the patients´ payment method to influence their compliance.

#### Decision-making and experience

There is lack of consensus among the informants as to how to approach and handle the PDHS guidelines for the CCS. The informants recall it to be more or less, widely known that clinicians with limited professional experience tend to follow the guidelines more closely than more experienced care providers. The latter use their experience to apply a more holistic approach to their decision-making. This tends to affect which patients are offered CCS enrolment. Factors such as compliance with self-care, financial stability, and the patient’s own commitment are essential to successful enrolment.

*“If the patients themselves are not already enroled or ask to be enroled, I am not sure I would offer it to them”.*

One of the informants with shorter work-life experience express the difficulty of matching the right treatment plan and risk assessment to the patient needs.

*“I need to see the patient several times to be able to stand by my decision. You may assess them to the wrong risk group if you do not know this person or if you only met them once before”.*

One informant addresses the differences in decision making as a dilemma as the patient overall oral health care maintenance could alter due to which caregiver they go to.

*“Cause if you choose to visit caregiver number 1 or number 4 you will receive different treatments, even though your health condition is the same. Nonetheless, if you attend another clinic”.*

#### Conducting risk assessments

Predicting a patient’s oral health progression is difficult; several different components need to be considered. The informants emphasize that they want to be compassionate when making assessments, especially if there is a risk that the patient might be assigned to a high risk-group. While the number of regular check-ups is based on the risk assessment and generated automatically, preventive dental care is not. Therefore, the informants must manually assess this need and frequency, this tends to result in recall intervals that are too short.

*“I believe it is easier to assess a higher and slightly more extensive prophylaxis program than the other way around. Since you are afraid to give insufficient care … you do not want them [the patients] to develop a progressive need for care”.*

One informant with longer experience within the PDHS recalls the several developmental changes that the risk assessment has gone through over the years.

*“When I started [as a dentist], you were supposed to visit the dentistry once every year. Then came the change toward a more individual recall process. At the beginning, each dentist made their own interpretation of the guidelines resulting in more or less unequal assessments. That resulted in computer algorithms which weren’t supposed to be changed which resulted in the system that we use today”.*

#### Charging patients for treatments

The informants feel more comfortable when treating the CCS patients, as payment is already taken care of. The payment procedure is otherwise regarded as a hindrance, taking up chairside time.

*“We should make revenue of x amount per hour, and then it is important to charge the patients to add up. It can be difficult sometimes, to charge for your own treatments. But with CCS it does not matter, I can use whatever procedure code I want. Yes, so it is completely different”.*

One treatment/method that is considered needed for patients´ compliance to self-care routines and oral health care knowledge is motivational interviewing. Although, it is known for the informants to be difficult to convince patients of its benefits compared to its costs.

*“Patients do not think of restorative treatments for cavities to be something they can choose to decline. They are likely to accept that treatment because they get something done during the visit”.*

### Reasons for inequality in access to oral care

Patient attendance within the dentistry differs between regions, but the informants also recall a differentiated accessibility between clinics. Recruitment of dental staff is easier in larger cities then rural clinics. This influences the provided care as well as the recalls and prioritization of patients.

#### Imbalance of patient volumes and dental staff resources

The informants experience different needs of patient volumes at their clinics. Some described an overload of patients, and they were unable to recall all patients within a reasonable time frame. This was considered stressful and even overwhelming. Additionally, some informants mentioned difficulty in complying with the PDHS regulation that all patients who have chosen to attend a specific clinic must be seen.

*“We [the PDHS] are the regional dental providers and should be available to all. This means that we cannot close the door on people seeking dental care at our clinics. But at the same time, we want to give good care, the patient should feel well taken care of and we ourselves should not be under too much pressure”.*

The capacity and resources of clinics are of highest importance when it comes to delivering dental care of good quality. One informant mentioned that two regions in the north of Sweden are considering phasing out the CCS due to unequal availability for the patients that is not enrolled. The dental hygienist’s professional role is highly needed within the CCS as many examinations and prophylactic treatments are allocated to them.

*“It is of utmost importance that there is a resource of dental hygienists, or else it would be too expensive [to pursue the CCS]. … then we need to increase costs to the extent that patients would decline the offered contract”.*

#### Recall procedures

A larger population of healthy individuals are prioritized in the recall system. One example mentioned is that free dental care for children and adolescents has been extended from 19 to 23 years of age. Informants recall differencing intervals for patient groups and some mention alternating changes being made to match the patient needs within the population.

*“For those who have stayed healthy throughout the years, we can probably extend the intervals from one year to two without seeing any impairments. Hopefully we then get more resources to see those who are in greater need for care”.*

Furthermore, with reference to recall appointments, the informants consider that they have a greater obligation to recall the CCS patients within the agreed interval, rather than the FFS patients.

*“It is because we have a written agreement with the capitation contract patient. … it is more of a legal thing than a question of need. That is just how it is done”.*

## Discussion

The results illustrate the risk for the CCS to be counterproductive and impair the oral health of un-enrolled patients unless its components are in balance. These components include organizational factors, political decisions, and the dental staff’s attitudes and compliance with guidelines.

### Financial gain

In this study, the informants appreciated the fact that CCS could enhance patient attendance and financial stability for both PDHS and patients. The informants found the financial aspect of enrolment difficult to present to patients and the CCS simplified this, shifting the focus from payments to treatments. Financial aspects would then be discussed only once every three years. This is in accordance with the government proposition of Award Dental Care, regarded as the theoretical forerunner of CCS [32]. It states that the CCS would provide cost control simplification as well as continuity of patient visits. This should also enhance the delivery of preventive dental care. However, this study shows that it may be difficult to assess the right level of preventive care. If the patients receive appropriate care at the appropriate time, they are more likely to retain their healthy oral status [33].

Rubin and Edelstein [33] claim that reliable and valid health outcome measures may be further evaluated for the traditional payment models within medicine and dentistry. They stress that these systems focus on volume of treatments but seem to overlook the actual outcome and value of delivered care. However, their view of capitation is based on care providers that gain directly from the FFS hence get incentives to more extensive and voluminous treatment plans. Furthermore, they claim that CCS induces a risk of undertreatment, as the care provider, rather than the Swedish PDHS, gains directly from the CCS. These observations are supported by Grönqvist [18] and Grytten [6]. Therefore, the incentives are linked to the company and not to the clinicians themselves. Our results can therefore be compared more readily with other Swedish studies with reference to incentives for treatment delivered and enrolment in the CCS. Internationally there is still a need for more research into delivery of dental care in relation to payment model [2].

In addition to the findings of Rubin and Edelstein [33], Holmes et al. [10] mentioned the risk of an overly productive dentistry in terms of achieving company enrolment numbers should be weight against the quality of performed dental care. In comparison to this, in the present study outcome, the informants reckon it sometimes to be difficult to charge for treatments in the FFS. This may also enhance the risk for undertreatments of patients with smaller economical marginals. Especially if they decline the recommended treatment that is offered. One informant claims the CCS to be most benefitable to higher risk group patients. However, these patients tend to decline the enrolment offer with the same reasoning, the cost is too high. In this aspect, the CCS simplifies that the right type of oral health care is performed. However, the CCS seems to be allocated to the healthier patients and functions to prevent future disease.

### Enrolment

The informants stressed that it could be difficult to achieve an equal balance of the risk assessment result between the PDHS and patients. The results support the concerns mentioned by Hallberg et al. [34]. The informants claim it to be generally known that patients in lower-risk groups are easier to enroll and that the PDHS gains from these patients. This is also confirmed by previous studies [14, 21] and the government proposition of awarded dental care in 1993, the theoretical forerunner of the CCS, mentioned this as a risk for inequality [32]. This is called ´cream skimming´ and could be avoided if the set fee could be differentiated according to patient age or through other risk assessment evaluations. This observation is in accordance with a study by Busby et al. [35] who also emphasized the importance of the patients’ age, as risk assessments are based on oral disease progression and previous dental care. Younger patients are therefore more likely than older patients to be enrolled in the CCS. The CCS then misses out on the so-called ´swings and roundabouts´ effect, meaning that ultimately the set fee should account for patients in both lower and higher risk groups. Previous studies mention enrolment as the patient´s own choice [14, 21]. The dental staff’s decision as to whether to offer enrolment at all is not considered. This might affect study results of specific patient characteristics in association with risk group, or CCS enrolments, as disclosed by the results of the present study. The risk assessment needs to be easy for the clinician to use in order to comply to company guidelines, make considerations based on his/her experience and patient compliance and acceptance to the treatment plan.

### Balance of treatment need and performed treatments

The risk assessment system used in the PDHS have previously been reviewed [36, 37] even though it seems like the guidelines resulting in the risk assessment is put together by the PDHS themselves according to Hänsel Petersson et al. [37]. They also conclude that the risk assessment used does not reach the expectations in sensitivity to distinguish patients with a low risk of dental caries to the ones with higher risk. Milosavljevic et al. [38] adds validity to this study results as they found that clinical judgments and the following treatments planned differed between care providers. This indicates that guidelines could add a collective standpoint on how to conduct assessments. Mejàre et al. [36] shows that the validity of risk assessments is limited and should be further investigated. There is a risk for overtreatment since most of the patients enrolling in the capitation system are in the lowest risk-group. There is also risk for under-treatment since patients in higher-risk groups may not receive the dental treatment that they need due to costs.

### Availability and patient attendance

The informants working in clinics with high patient pressure regarded this as overwhelming, the PDHS requirement that all patients applying to their clinic should be accepted. They were concerned that this would affect both their well-being and work satisfaction. This is in accordance with earlier research [39]. Accessibility is also a barrier, because of the discrepancies in the availability of dental staff. This was acknowledged by the Swedish government commission on Equal Dental Health presented in March 2021 [40], highlighting the importance of adjusting for socioeconomic factors which could contribute to inequality of dental health within the population. Of relevance in this context is the ´inverse care law´ addressing general health, presented by Hart in 1971 [41]: proposing that resources were more likely to be distributed to and aligned with the healthier section of society rather than the less healthy. Dharamsi & McEntee [27] address this as a question of distributive justice. They discuss the importance of a dental health care service that balance the principles of social justice and accessibility for the population and a reasonable and fair allocation of resources for dentistry.

In the present study, apart from dental personnel selecting which patients should be invited to enroll, it was ultimately up to the patients to decide whether to accept the enrolment offer. He [42] reported the same associations in a study of intentions of citizens of Hong Kong to enrol in a new private health insurance scheme. The young, well-educated healthy patients were more likely to choose private insurance. This group of patients had the advantage of being satisfied with their health care but more importantly, of being able to make this choice even though the costs were higher. In contrast, the patients with poor health showed a heightened interest in enroling as the waiting time for specialist care was shorter. This is of interest when viewing the CCS system in Sweden. Common factors in the present study are for example patient characteristics. The informants thought that the less healthy patients might benefit more from enrolment than the healthy ones. In future studies it would be of interest to evaluate the reasons underlying these experiences and the clinicians´ decision-making that leads to the enrolments.

## Strengths and limitations

The informants comprised dentists and dental hygienists who advised their clinic manager that they were interested in participating in this study. It was anticipated that the informants would contribute on the basis of their work experience and divergent opinions on the subject. A strength of the study was that it included both dentists and dental hygienists, which might give a more comprehensive view of the topic. The author (EB) has followed the analysis process according to Charmaz [28] and consulted with her co-authors, increasing the reliability and credibility of the results. The number of informants were chosen according to GT procedure and we reached saturation after 11. This means that no new insights were added with further interviews and that the data would not add to the already existing patterns of findings.

It would have been preferable to include two more counties in Sweden to achieve a more national perspective of the results, but the invitation to participate was declined. Due to travel restrictions imposed by the Covid-19 pandemic, all interviews were conducted online. This could have influenced the results because the informants were unfamiliar with digital communication. To minimize this effect, the author held discussions with the informants before the interviews, in order to create a more comfortable situation. It is anticipated that this means of communication will become more common as the pandemic continues. Moreover, it is more cost-efficient.

## Conclusions

This study showed that the informants favoured CCS, experiencing appointments with these patients to be less constrained than with FFS patients. However, they see a need for balanced resources, or else there is a risk for CCS to be counterproductive and may impair the oral health of un-enrolled patients. Also highlighted was the conflict of loyalty experienced by the informants in assuming the roles of both insurance sales agent and care provider. Moreover, the informants were aware that their subjectivity influences which patients are offered enrolment.
